# Seroprevalence and factors associated with hepatitis B infection among the hill tribe adult population in Thailand: a cross-sectional study

**DOI:** 10.1186/s12879-020-05221-1

**Published:** 2020-07-10

**Authors:** Panupong Upala, Tawatchai Apidechkul, Ratipark Tamornpark, Chalitar Chomchoei, Fartima Yeemard

**Affiliations:** 1 Center of Excellence for The Hill tribe Health Research, Mae Fah Luang University, Chiang Rai, Thailand; 2 School of Health Science, Mae Fah Luang University, 333 Mo.1 Tasud Sub-district, Muang District, Chiang Rai, 57100 Thailand; 3 Chulabhorn Royal Academy, Bangkok, Thailand

**Keywords:** Seroprevalence, Hepatitis B virus, Factor associated, Hill tribe, Adults

## Abstract

**Background:**

Hepatitis B virus (HBV) infection is one of the greatest public health burdens, particularly for people living with several barriers to access to health care services, such as the hill tribe adult population in Thailand. People aged 25 years and over who are out of the target population for HBV immunization under the national Expanded Program on Immunization (EPI) are at risk of HBV infection. The study aimed to estimate the prevalence and determine the factors associated with HBV infection among hill tribe adults aged 25 years and over living in Chiang Rai Province, Thailand.

**Methods:**

A cross-sectional study design was used to collect information on hill tribe adults aged 25 years and over living in 36 selected hill tribe villages in Chiang Rai Province. All people living in the selected villages who met the criteria were invited to participate in the study. A validated questionnaire and a 5-mL blood specimen were used as research instruments. Hepatitis B surface antigen (HBsAg), antibody to hepatitis B surface (anti-HBs), and antibody to hepatitis B core (anti-HBc) were detected by using the Wondfo Test Kit^@^, which has high sensitivity and specificity. Logistic regression was used to detect the associations between variables at the significance level of α = 0.05.

**Results:**

A total of 1491 individuals were recruited into the analysis; 60.8% were females, 81.3% were aged between 30 and 60 years, and 86.0% were married. The majority were illiterate (54.9%), were Buddhist (55.7%), worked in agricultural sectors (87.3%), and had an annual income of less than 50,000 baht per year (72.9%). The overall prevalence of hepatitis B infection was 26.6%; 7.6% were positive for HBsAg, 19.2% were positive for anti-HBs, and 18.9% were positive for anti-HBc. In the multivariate analysis, three variables were found to be associated with hepatitis B infection: those who were in the Yao and Lisu tribes had a 1.64-fold (95% CI = 1.08–2.49) and a 1.93-fold (95% CI = 1.10–3.31) greater chance, respectively, of HBV infection than did those in the Karen tribe; those who were Christian had a 1.41-fold (95% CI = 1.06–1.87) greater chance of HBV infection than did those who were Buddhist; and those who did not use alcohol had a 1.29-fold (95% CI = 1.01–1.65) greater chance of HBV infection than did those who used alcohol.

**Conclusions:**

It is necessary to develop and implement effective public health interventions among hill tribe adult populations who are not part of the EPI-targeted population, particularly Christians, those in the Lisu and Yao tribes, and those who do not use alcohol, to reduce the HBV infection rate, save lives and reduce medical expenses.

## Background

Hepatitis B virus infection is the greatest infectious disease in the human population, with approximately 257 million people living with chronic hepatitis B infection, which is defined by HBsAg positivity globally [1]. It is a common infectious disease that is transmitted person-to-person during delivery [2] and through contaminated blood [3] and other body fluids [2]. The targeted organ of the infection is the human liver [4]. Afterward, wide ranges of pathogenesis and complications could occur to adversely affect the infected liver’s health [5]. The serious final stage of infection is cancer, which mostly presents as aggressive progression for the organ with a very poor prognosis [6]. The WHO estimated that 887,000 deaths are reported every year from cirrhosis and hepatocellular carcinoma resulting from hepatitis B infection [1]. The WHO also reported that the highest prevalence of hepatitis B infection was in the Asia Pacific region (6.2%) [1]. A total of US$58.7 billion is needed to address hepatitis among 67 low- and middle-income countries by 2030, which could prevent 90.0% of new cases of infection and save the lives of 65.0% of those with existing cases of infection, including individuals in Thailand [7].

Thailand reported 2.2–3 million people who were hepatitis B carriers and who were HBsAg positive [8]. Thailand has included the hepatitis B vaccine on one of the lists in the Expended Program on Immunization (EPI) for almost 25 years since it was first implemented in 1992. Since then, this program has reduced the HBV carrier rate among children aged younger than 25 years by less than 1.0%. However, a high prevalence of those aged 25 years and older were still reported to be carriers of hepatitis B, with an average of 5.9% [9]. The hepatitis B vaccine has significantly reduced the number of hepatitis carriers and other medical expenses and has been used for treatment and care related to hepatitis B viral infection in the Thai population. However, this does not mean that all Thai people can access health care services equality, especially immunizations for children and other targeted populations under the national EPI, even if they do not need to pay a fee [10]. In addition, those who do not fall within the target of the Thai national EPI are requested to pay for US$70 for each HBV vaccine (three standard doses) [11]. Some Thai people live in poor economic conditions, have low levels of education and face several barriers to access to medical services, such as language, distance, and stigmatization [12].. The hill tribe people are a good example of those with atypical access to Thai medical services, since they are living in rural areas and have specific cultures and perceptions, particularly with unrespect to access to medical services, including immunization for children [13–15].

The hill tribe people migrated from southern China and settled down along the borders of Thailand-Myanmar-Laos over a couple centuries [16]. They are now composed of six main tribes: Akha, Lahu, Hmong, Yao, Karen, and Lisu [16]. In 2019, approximately 2.5–3.5 million people lived in Thailand [15], and 250,000–350,000 people lived in Chiang Rai Province [17]. Approximately 70.0% [18] of the hill tribe people have Thai identification cards (IDs), which are used for access to all public services, including health care services [19].

Unfortunately, the hill tribe people are living in the era of HBV vaccine implementation in Thailand, but the prevalence estimates of hepatitis B surface carrier (HBsAg) and the total hepatitis B infection were reported to be higher among the hill tribe people than in the Thai population in the same age category at 10.3% [20]. With the specific history of those in the hill tribes who migrated from China, which has the highest prevalence of hepatitis B infection in the world [21], and given these individuals’ very limited access to health care services, the hill tribe population in Thailand is at risk of hepatitis B infection. Moreover, lifestyles and practices such as ear piecing [22], high alcohol consumption [23] and other substance use, including drug injection by untrained health professionals [24], all while living far away from the city, could lead individuals to become vulnerable to hepatitis B infection.

There is no scientific information regarding hepatitis B infection in the hill tribe population aged 25 years and older before the generation of the hepatitis B vaccine implementation in Thailand. Thus, the study aimed to estimate the prevalence of hepatitis B infection by detecting hepatitis B surface antigen (HBsAg), antibody to hepatitis B surface (anti-HBsAg), and total antibody to hepatitis B core (anti-HBc) according to the standard guidelines of the Centers for Disease Control and Prevention (CDC, US) [25].

## Methods

### Study design

A community-based cross-sectional study was used to collect information from the participants.

### Study settings

The study was conducted in the hill tribe villages in Chiang Rai Province, Thailand. There are six main hill tribes living in the study settings: Akha, Lahu, Hmong, Yao, Karen, and Lisu. In 2019, there were 749 hill tribe villages in Chiang Rai, which included 316 Lahu villages, 243 Akha villages, 63 Yao villages, 56 Hmong villages, 36 Karen villages, and 35 Lisu villages [17]. Six villages in each tribe were randomly selected to be study settings from the lists of the hill tribe villages in 2019 [17]; therefore, 36 hill tribe villages located in 14 districts in Chiang Rai Province served as the study settings [17].

### Study population

The study population included hill tribe people who lived in the selected villages and met the eligibility criteria. The eligibility criteria for the study were as follows: i) belonging to one of the six main hill tribes in Thailand, ii) able to provide essential information according to the study protocol, and iii) aged 25 years and over.

### Study sample and sample size calculation

The sample size was calculated from the standard formula of a cross-sectional study (Z = 1.96, *P* = 0.13 [20], Q = 0.87 and e = 0.05); therefore, at least 246 participants in each tribe were required for the analysis. Thus, a minimum of 1475 participants were needed in the whole study.

### Research instruments and their development

The questionnaire was developed from the literature review and was discussed with the medical staff working in an infectious diseases clinic at the Chiang Rai Regional Hospital (a tertiary hospital). The content validity of the questionnaire was also determined by the item-objective congruence (IOC) technique by three external experts (one epidemiologist, one public health specialist, and one infectious diseases physician), who provided their opinions regarding the relevance of the questions and the content of the study. The questions scoring less than 0.50 were excluded from the questionnaire set, those scoring between 0.51–0.70 were revised as comments obtained by the experts, and those scoring equal or more than 0.71 were included in the set of questionnaires.

Afterwards, the feasibility and ordering of the questions on the questionnaire were determined through its piloting in 15 samples with characteristics similar to those of the study participants at Mae Chan Hospital, Chiang Rai Province, Thailand. After the questionnaire was revised following the pilot test, the final questionnaire consisted of three parts. Part one included 10 questions that were used to collect the sociodemographic characteristics of the participants, such as age, sex, religion, marital status, occupation, and income. Part two included 14 questions that were used to collect information on risk behaviors related to hepatitis B infection, such as alcohol use, methamphetamine use, tattooing, history of blood transfusion, history of organ transplantation, and history of hepatitis B vaccination. Three open-ended questions were provided to collect laboratory results.

### Data collection procedure

After approval was obtained for the research ethical consideration, development and completion of the questionnaire, all 14 relevant district government officers were contacted by the researchers to obtain their approval for access to the villages. Afterward, village headmen were contacted and were provided with details on the study. Targeted populations who met the criteria were informed regarding the execution of the study and made appointments for data collection 3 days prior.

On the date of data collection, all participants were asked to obtain the informed consent form after providing all essential information for the study, followed by 5-mL blood specimens being drawn by licensed health professionals. The questionnaire was completed by self-administration in a private and confidential room. Those who could not understand Thai received assistance in completing the questionnaire by village public health volunteers who were fluent in both Thai and local languages. All processes lasted for 30 min each.

### Laboratory methods

Five-milliliter blood specimens were drawn from all participants. The laboratory analysis for hepatitis B markers HBsAg, anti-HBs, and total anti-HBc was performed at the Mae Fah Luang Central Medical Laboratory on the same day that blood specimens were drawn by licensed medical technicians.

The Wondfo One Step HBsAg Serum/Plasma^@^ was used for the detection of HBsAg. It has 96.2% sensitivity and 99.3% specificity. The Wondfo Diagnostic Kit^@^ was used for the detection of anti-HBs and total anti-HBc, with 97.3% sensitivity and 99.2% specificity.

### Outcomes

Hepatitis B infection was defined based on the standard guideline for the interpretation of hepatitis B serologic test results of the Centers for Disease Control and Prevention (CDC), United States [25], such that those who were positive for either or both HBsAg and anti-HBs, except for those who had a negative anti-HBc, were considered to have hepatitis B infection.

### Statistical analysis

Data were double-entered into an Excel sheet and checked for errors before being transferring into SPSS program version 24 (SPSS, Chicago, IL) for analysis. Continuous and categorical data were described properly to present the general characteristics of the participants and their behaviors, including hepatitis B biomarkers. Logistic regression was used to identify the factors associated with hepatitis B infection at the significance level of α = 0.05.

## Results

A total of 1491 individuals were recruited into the analysis; 60.8% were females, 81.3% were aged between 30 and 60 years, and 86.0% were married. The majority were illiterate (54.9%), were Buddhist (55.7%), worked in agricultural sections (87.3%), and had an annual income of less than 50,000 baht per year (72.9%). A large proportion held Thai ID cards (95.2%) and had the right to access to free-of-charge care (95.0%) (Table 1).

**Table 1:** General characteristics of participants

Characteristic	Number	Percent
**Total**	1491	100.0
**Sex**		
Male	585	39.2
Female	906	60.8
**Age** (years)		
< 30	66	4.4
30–60	1212	81.3
> 60	213	14.3
*Mean = 48.9 years, SD = 11.0, Min = 25, Max = 78*		
**Marital status**		
Single	73	4.9
Married	1282	86.0
Ever married	136	9.1
**Education**		
Illiterate	819	54.9
Primary school	383	25.7
Secondary school	217	14.6
High school and above	72	4.8
**Religion**		
Buddhist	830	55.7
Christian	661	44.3
**Tribe**		
Akha	295	19.8
Lahu	346	23.2
Hmong	299	20.1
Yao	167	11.2
Lisu	68	4.6
Karen	316	21.2
**Having Thai ID card**		
Yes	1420	95.2
No	71	4.8
**Occupation**		
Farmer	1302	87.3
Working in a trade	36	2.4
Daily wage employee	153	10.3
**Right to access free health care services**		
Thai universal health scheme	1361	91.3
State enterprise officer	15	1.0
Social security scheme	40	2.7
Private insurance	4	0.3
No	71	4.8
**Number of family members** (persons)		
< 4	725	48.6
4–7	596	40.0
> 7	170	11.4
**Annual income** (baht)		
< 10,000	158	10.6
10,001-50,000	929	62.3
> 50,000	404	27.1

One-third of the participants used alcohol (37.8%), but a few people used other substances: amphetamine (3.4%), heroin (0.5%), opium (3.4%), and marijuana (2.1%). More than half of the participants had experienced ear piercing (53.1%), and 11.3% were tattooed. A small proportion of individuals had experienced blood transfusion (3.0%), organ transplantation (1.5%), medical surgery (11.1%), and acupuncture (7.6%). Five (5) variables were found the statistical significances on the proportions in different health behaviors between sex; tattooed (*p*-value< 0.001), ear piercing (p-value< 0.001), history of medical surgery (p-value = 0.017), drug injection from unqualified health professional (p-value = 0.044), and sharing toothbrush p-value = 0.001) (Table 2).

**Table 2:** Identifications of health behaviors by sex

Health behaviors	Total		Male		Female		χ^**2**^ *(p-value)*
	n	%	n	%	n	%	
**Alcohol use**							
No	927	62.2	370	63.2	557	61.5	0.47 *(0.492)*
Yes	564	37.8	215	36.8	349	38.5	
**Methamphetamine use**							
No	1441	96.6	567	96.9	874	96.5	0.23 *(0.634)*
Yes	50	3.4	18	3.1	32	3.5	
**Heroin use**							
No	1484	99.5	581	99.3	903	99.7	0.95 *(0.331)*
Yes	7	0.5	4	0.7	3	0.3	
**Opium use**							
Yes	1440	96.6	559	95.6	881	97.2	3.01 *(0.080)*
No	51	3.4	26	4.4	25	2.8	
**Marijuana use**							
Yes	1460	97.9	571	97.6	889	98.1	0.47 *(0.495)*
No	31	2.1	14	2.4	17	1.9	
**Tattooed**							
No	1317	88.3	464	79.3	853	94.2	75.88 *(< 0.001*^a^*)*
Yes	174	11.7	121	20.7	53	5.8	
**Ear piercing**							
No	699	46.9	504	86.2	195	21.5	596.26 *(< 0.001*^a^*)*
Yes	792	53.1	81	13.8	711	78.5	
**History of blood transfusion**					881	60.9	
No	1446	97.0	565	96.5	881	97.2	0.53 *(0.467)*
Yes	45	3.0	20	3.4	25	2.8	
**History of organ transplantation**							
No	1468	98.5	573	97.9	895	98.8	1.64 *(0.200)*
Yes	23	1.5	12	2.1	11	1.2	
**History of medical surgery**							
No	1325	88.9	534	91.3	791	87.3	5.68 *(0.017*^a^*)*
Yes	166	11.1	51	8.7	115	12.7	
**Drug injection from unqualified health professional**							
No	1442	96.7	559	95.6	883	97.5	4.06 *(0.044*^a^*)*
Yes	49	3.3	26	4.4	23	2.5	
**Acupuncture**							
No	1377	92.4	544	93.0	833	91.9	0.55 *(0.457)*
Yes	114	7.6	41	7.0	73	8.1	
**Sharing toothbrush**							
No	1384	92.8	527	90.1	857	94.6	10.84 *(0.001*^a^*)*
Yes	107	7.2	58	9.9	49	5.4	
**History of receiving HBV vaccine**							
Yes	78	5.2	113	19.3	140	15.5	4.55 *(0.208)*
No	1059	71.0	313	53.5	515	56.8	
Not sure	192	12.9	105	17.9	175	19.3	
Don’t know	162	10.9	54	9.2	76	8.4	

^a^Significance level at α = 0.05

Among the 1491 participants, 113 (7.6%) were positive for HBsAg, 19.2% were positive for anti-HBs, and 18.9% were positive for anti-HBc. The overall prevalence of hepatitis B infection was 26.6% (396 out of 1489). Moreover, three participants were positive for both anti-HBsAg and HBsAg. There were no statistical differences among the tribes on HBsAg, anti-HBs, and total anti-HBc status (Table 3).

**Table 3:** Hepatitis B biomarkers in different tribes

Biomarkers	Total		Akha		Lahu		Hmong		Yao		Lisu		Karen		χ^**2**^ *(p-value)*
	n	%	n	%	n	%	n	%	n	%	n	%	n	%	
**HBsAg**															
Positive	113	7.6	15	5.1	26	7.5	22	7.4	20	12.0	9	13.2	21	7.6	10.7 *(0.056)*
Negative	1378	92.4	280	94.9	320	92.5	277	92.5	147	88.0	59	86.8	295	18.7	
**Anti-HBs**															
Positive	286	19.2	66	22.4	59	17.1	49	16.4	37	22.2	16	23.5	59	20.6	6.29 *(0.278)*
Negative	1205	80.8	229	77.6	287	82.9	250	83.6	130	77.8	52	76.5	257	81.3	
**Total anti-HBc**															
Positive	283	18.9	66	22.4	58	16.7	48	16.0	37	22.2	16	23.5	58	18.4	7.07 *(0.215)*
Negative	1208	81.1	229	77.6	288	83.3	251	84.0	130	77.8	52	76.5	258	81.6	
**HBsAg-positive and anti-HBs-positive**	3	0.2	0	0.0	1	33.3	1	33.3	0	0.0	0	0.0	1	33.3	N/A
**HBV infection**	396	26.6	81	20.5	84	21.2	70	17.7	57	14.4	25	6.3	79	19.9	N/A

Seven (7) variables were found the statistical significance in having hepatitis B infection; age (*p*-value = 0.021), tribe (p-value = 0.042), education (p-value = 0.006), occupation (p-value = 0.031), income (p-value = 0.008), history of having hepatitis B infection in family member (p-value = 0.049), and alcohol use (p-value = 0.042) (Table 4).

**Table 4:** Univarite and multivariate analyses in identification factors associated with hepatitis B infection

Factors	Hepatitis B infection		χ^**2**^ *(p-value)*	OR	95% CI	***p***-value	OR_**Adj**_	95% CI	***p***-value
	Yes n (%)	No n (%)							
**Sex**									
Male	150 (25.6)	435 (74.4)	0.42 *(0.519)*	0.93	0.73–1.17	0.519			
Female	246 (27.2)	660 (72.8)		1.00					
**Age** (years)									
< 30	18 (27.3)	48 (72.3)	7.72 *(0.021*^a^*)*	1.00					
30–60	338 (27.9)	874 (72.1)		1.03	0.59–1.80	0.914			
> 60	40 (30.1)	173 (69.9)		0.62	0.33–1.17	0.140			
**Tribe**									
Akha	81 (27.5)	214 (72.5)	11.50 *(0.042*^a^*)*	1.14	0.79–1.63	0.490	1.08	0.73–1.59	0.694
Lahu	84 (24.3)	262 (75.7)		0.96	0.68–1.37	0.829	0.91	0.62–1.33	0.623
Hmong	70 (23.4)	229 (76.6)		0.92	0.63–1.33	0.646	0.99	0.67–1.46	0.966
Yao	57 (34.1)	110 (65.9)		1.56	1.03–2.34	0.034^a^	1.64	1.08–2.49	0.020^a^
Lisu	25 (36.8)	43 (63.2)		1.74	1.00–3.04	0.049^a^	1.93	1.10–3.41	0.023^a^
Karen	79 (25.0)	237 (75.0)		1.00			1.00		
**Marital status**									
Married	18 (24.7)	55 (75.3)	5.42 *(0.066)*	1.00					
Single	353 (27.5)	929 (72.5)		1.16	0.67–2.01	0.592			
Ever married	25 (18.4)	111 (81.6)		0.69	0.35–1.37	0.286			
**Religion**									
Buddhism	205 (24.7)	625 (75.3)	3.32 *(0.068)*	1.00			1.00		
Christianity	191 (28.9)	470 (71.1)		1.24	1.01–1.56	0.049^a^	1.41	1.06–1.87	0.017^a^
**Education**									
Illiterate	198 (24.2)	621 (75.8)	12.28 *(0.006*^a^*)*	0.83	0.48–1.42	0.496			
Primary school	100 (26.1)	283 (73.9)		0.92	0.52–1.62	0.768			
Secondary school	78 (35.9)	139 (64.1)		1.46	0.81–2.62	0.206			
High school and above	20 (27.8)	52 (72.2)		1.00					
**Having Thai ID card**									
Yes	373 (26.3)	1047 (73.7)	1.30 *(0.254)*	1.00					
No	23 (32.4)	48 (67.6)		1.35	0.81–2.24	0.156			
**Having free access to health care services**									
Yes	376 (26.5)	1044 (73.5)	0.10 *(0.753)*	1.00					
No	20 (28.2)	51 (71.8)		0.92	0.54–1.56	0.761			
**Occupation**									
Farmer	345 (24.5)	957 (73.5)	6.97 *(0.031*^a^*)*	1.00					
Working in a trade	16 (44.4)	20 (55.6)		2.22	1.14–4.33	0.019^a^			
Daily wage employee	35 (22.9)	118 (77.1)		0.82	0.55–1.22	0.335			
**Number of family members** (persons)									
< 4	202 (27.9)	523 (72.1)	1.39 *(0.499)*	1.00					
4–7	153 (25.7)	443 (74.3)		0.89	0.70–1.14	0.372			
> 7	41 (24.1)	129 (75.9)		0.82	0.56–1.21	0.324			
**Income** (baht)									
< 10,000	45 (28.5)	113 (71.5)	9.65 *(0.008*^a^*)*	1.00					
10,001 – 50,000	222 (23.9)	707 (76.1)		0.78	0.54–1.15	0.217			
> 50,000	129 (31.9)	275 (68.1)		1.18	0.79–1.76	0.427			
**History of having hepatitis B vaccination**									
Yes	65 (25.7)	188 (74.3)	3.52 *(0.318)*	1.00					
No	227 (27.4)	601 (72.6)		1.09	0.79–1.51	0.589			
Not sure	78 (27.9)	202 (72.1)		1.12	0.76–1.64	0.573			
Don’t know	26 (20.0)	104 (8.0)		0.72	0.43–1.21	0.216			
**History of having hepatitis B infection in family member**									
Yes	24 (30.8)	54 (29.2)	7.84 *(0.049*^a^*)*	1.00					
No	278 (26.3)	781 (72.7)		0.80	0.49–1.32	0.384			
Not sure	62 (32.3)	130 (67.7)		1.07	0.61–1.89	0.808			
Don’t know	32 (19.8)	130 (80.2)		0.55	0.29–1.03	0.061			
**Tattooed**									
No	345 (26.2)	972 (73.8)	0.76 *(0.382)*	1.00					
Yes	51 (29.3)	123 (70.7)		1.17	0.82–1.66	0.382			
**Ear pierced**									
No	183 (26.2)	516 (73.8)	0.10 *(0.756)*	1.00					
Yes	213 (26.9)	579 (73.1)		1.04	0.82–1.31	0.756			
**History of blood transfusion**									
No	384 (26.6)	1062 (73.4)	0.00 *(0.987)*	1.00					
Yes	12 (26.7)	33 (73.3)		1.01	0.51–1.97	0.987			
**History of organ transplantation**									
No	391 (26.6)	1077 (73.4)	0.28 *(0.598)*	1.00					
Yes	5 (38.5)	18 (61.5)		0.77	0.28–2.08	0.599			
**History of medical surgery**									
No	350 (26.4)	975 (73.6)	0.13 *(0.722)*	1.00					
Yes	46 (27.7)	120 (72.3)		1.07	0.74–1.53	0.722			
**Receiving a drug injection from an untrained medical professional**									
No	387 (26.8)	1055 (73.2)	1.74 *(0.187)*	1.00					
Yes	9 (27.4)	40 (72.6)		0.61	0.30–1.28	0.191			
**Having acupuncture**									
No	363 (26.4)	1014 (73.6)	0.36 *(0.548)*	1.00					
Yes	33 (28.9)	81 (71.1)		1.14	0.75–1.74	0.548			
**Sharing toothbrush**									
No	365 (34.6)	1019 (65.4)	0.34 *(0.558*)	1.00					
Yes	31 (28.9)	76 (71.1)		1.14	0.74–1.76	0.558			
**Alcohol use**									
Yes	133 (23.6)	431 (76.4)	4.12 *(0.042*^a^*)*	1.00			1.00		
No	263 (28.4)	664 (71.6)		1.28	1.01–1.63	0.043^a^	1.29	1.01–1.65	0.040^a^
**Methamphetamine use**									
Yes	11 (22.0)	39 (78.0)	0.55 *(0.458)*	0.77	0.39–1.53	0.459			
No	385 (26.7)	1056 (73.3)		1.00					
**Heroin use**									
Yes	3 (42.9)	4 (57.1)	0.96 *(0.328*)	2.08	0.46–9.34	0.338			
No	393 (26.5)	1091 (73.5)		1.00					
**Opium use**									
Yes	13 (54.5)	38 (74.5)	0.03 *(0.860)*	0.94	0.50–1.79	0.860			
No	383 (26.6)	1057 (73.4)		1.00					
**Marijuana use**									
Yes	5 (16.1)	26 (83.9)	1.77 *(0.184)*	0.53	0.20–1.38	0.191			
No	391 (26.8)	1069 (73.2)		1.00					

^a^Significance level at α = 0.05

In the univariate analysis, five variables were found to be associated with hepatitis B infection: tribe, religion, occupation, income, and alcohol use. However, in the multivariate analysis, after controlling for sex, age, marital status, education, and ID card, only three (3) variables remained associated with hepatitis B infection: tribe, religion, and alcohol use. Those who were in the Yao and Lisu tribes had a 1.64-fold (95% CI = 1.08–2.49) and a 1.93-fold (95% CI = 1.10–3.41) greater chance, respectively, of HBV infection than did those in the Karen tribe. Those who were Christian had a 1.41-fold (95% CI = 1.06–1.87) greater chance of HBV infection than did those who were Buddhist. Those who did not use alcohol had a 1.29-fold (95% CI = 1.01–1.65) greater chance of HBV infection than did those who used alcohol (Table 4).

## Discussion

The hill tribe people aged 25 years and over who do not fall in the targeted area of HBV vaccination under the Thailand national EPI program have a high prevalence of HBV infection (26.6%); this population also has a high prevalence of HBsAg carriers (7.6%). The prevalence has increased according to the increase of age and income. Several risk behaviors affect the hill tribe population, including the use of various substances that could lead to HBV infection. However, individuals in certain tribes (Yao and Lisu), Christians and those who did not use alcohol were at a greater risk of HBV infection than others were.

The prevalence of HBV infection among the hill tribe adult populations was 26.6%, and the hepatitis B surface carrier rate was 7.6%, which are greater than the rates of these makers among the hill tribe aged 15–24 years, 3.0, 10.2, and 8.1%, respectively [20]. This might be the impact of the HBV immunization implementation in Thailand which has been introduced since 1992 [26, 27]. The hepatitis B markers are greater that the corresponding rates found in a study conducted among the elderly population in northeastern Thailand [28]. A systematic review of the hepatitis B infection rate in Thailand also reported that the overall prevalence rate was 5.1% [29], which is lower than the prevalence in the hill tribe adult population. A study in Vietnam [30] reported a prevalence rate of 9.0% of HBV, which was close to the rate among hill tribe adults in Thailand. The prevalence has increased according to age which is consisted with a study in China, [31, 32].

It is highly recommended that the HBV vaccine be implemented in high-prevalence areas by the United States Advisory Committee on immunization practice [27]. Therefore, Thailand needs to consider hepatitis B vaccine implementation in the hill tribe adult population in Thailand, the benefits of which have been clearly demonstrated by the major economic gains realized from HBV immunization in Italy [33].

Another interesting point from the study is that 0.2% (3) of participants were reported to be positive for both HBsAg and anti-HBs among the hill tribe adults. This was explained by Lada et al. [34], who determined the influence of some determinant variants from viral mutations during chronic infection in the host organ.

Some tribes had a greater chance of hepatitis B virus infection than did others; for example, individuals from the Lisu or Yao tribes were at a greater risk of HBV infection than were those from the Karen tribe. This finding coincides with the findings of a study in Iran [35], which reported that some tribes living in rural areas were at a significantly greater risk of HBV than others were. Moreover, a study in Malaysia [36] also reported that individuals in some tribes in Malaysia had a rate of HBV infection that was higher than the national rate. Another study conducted in India [37] reported that different tribes who were living in eastern India had significantly different rates of HBV infection. A study in the United States [38] also demonstrated that some groups of people had a greater risk of HBV infection than others did. Apidechkul [20] also confirmed that some hill tribe youths had a greater chance of HBV infection than others did. While having looked more closely into some behaviors such as alcohol use, tattooed, and ear piecing, it was found that the Lisu (20.6%) had a greater proportion of having tattooed than the Karen (17.7%) and Yao (9.6%), but the Yao (67.1%) had a greater proportion of having ear piecing than the Karen (59.8%) and Lisu (39.7%). These might be the potential risk factors of hepatitis B infection among the Lisu and Yao.

In the hill tribe population, adults who were Christians had a greater risk of hepatitis B infection than did those who were Buddhists. A study in Pakistan [39] reported that in some cultures, a reliance on religion may serve as a barrier to obtaining HBV vaccines, resulting in HBV infection. A study among Nigerian pregnant women [40] found that those following different religions had significantly different rates of HBV infection. In Thailand there is no evidence presented on restriction to access EPI program based on their religion. However, after taking a closer look into some variables in the participants’ background, it was found that those who were Christians had a lower education that those who were Buddhists significantly (*p*-value< 0.001). This might be making them access to EPI program in different proportions, and resulting to have different risks of hepatitis B infection. This association was supported by a study in China [41] and Malaysia [42].

Among the hill tribe adults, those who did not use alcohol had a greater risk of HBV infection than did those who used alcohol. This finding is not consistent with that of a study conducted in Korea [43], which clearly presented that those who had low levels of education tended to consume alcohol and develop HBV infection later. There are few studies on the association between alcohol use and HBV infection, but there are clear explanations for the association between alcohol use among those with hepatitis and the development of cirrhosis and hepatocellular carcinoma. Alcohol use might not the main route of HBV transmission among the hill tribe people in Thailand.

In our study, no participants refused to take part in the study. All participants who were positive for HBV biomarkers were informed to visit a medical doctor for proper treatment and care. Moreover, those who had a negative result for all HBV biomarkers were also asked to obtain the HBV vaccine from a health care provider properly and in a timely manner. The pattern of assessing exposures and outcomes in the same time on a cross-sectional study, it could impact the associations between variables while making interpretations [44]. Therefore, to make more clear on these associations, further stronger study designs to work out is recommended.

## Conclusion

The hill tribe adult population aged 25 years and over with HBV who have never been immunized under the Thai national EPI program have low levels of education, live in poor economic conditions and reside in areas of Thailand with a high prevalence of hepatitis B virus. They are highly vulnerable to hepatitis B infection, particularly those who are not using alcohol, Christians and those belonging to certain tribes, namely, the Lisu and Yao tribes. Effective public health interventions, including providing this population with HBV vaccines, need to be considered and implemented to reduce the HBV infection rate, save lives and decrease other medical costs.

## Data Availability

Data file is attached as supplement file. Additional data supporting these findings are available by personal request at Tawatchai.api@mfu.ac.th.

## Abbreviations

anti-HBc: Hepatitis B core antibody
anti-HBs: Hepatitis B surface antibody
CDC: Centers for Disease Control and Prevention
CI: Confident interval
EPI: Expended Program on Immunization
HBV: Hepatitis B virus
HBsAg: Hepatitis B surface antigen
ID: Identification card
IOC: Item-Objective Congruence
SD: standard deviation
WHO: World Health Organization
